# pH-Responsive Hybrid Hydrogels as Antibacterial and Drug Delivery Systems

**DOI:** 10.3390/polym10060660

**Published:** 2018-06-13

**Authors:** Shabnam Sattari, Abbas Dadkhah Tehrani, Mohsen Adeli

**Affiliations:** Department of Chemistry, Faculty of Science, Lorestan University, Khorramabad P.O. Box 465, Iran; sattari_sh@yahoo.com (S.S.); a_dadkhahtehrani@yahoo.com (A.D.T.)

**Keywords:** graphene, hydrogels, interpenetrating polymer network, drug delivery, antibacterial

## Abstract

This study describes the design and synthesis of organic–inorganic hybrid hydrogels based on an interpenetrating polymer network (IPN) composed of polyaspartic acid crosslinked by graphene nanosheets as the primary network and poly(acrylamide-co-acrylic acid) as the secondary network. Silver, copper oxide, and zinc oxide nanoparticles were formed within the gel matrix, and the obtained hydrogel was applied to a load and controlled release of curcumin. The loading of curcumin and the release of this drug from the gels depended on the nanoparticle’s (NP’s) content of hydrogels as well as the pH of the medium. The synthesized hydrogels showed antibacterial activity against *E. coli* and *S. aureus* bacteria. The ability of the synthesized hydrogels to incapacitate bacteria and their loading capacity and controlled release of curcumin qualify them for future therapies such as wound-dressing applications.

## 1. Introduction

In recent years, a variety of nanostructures has been developed to improve the therapeutic efficiency of drugs [1,2]. Compared to other delivery systems, polymeric hydrogels have been extensively used for this purpose. A polymer hydrogel has the capacity to swell and retain a large fraction of water within its structure that arises mainly from the presence of hydrophilic groups [1,2,3]. Additionally, hydrogels can control drug release due to changes (swelling, dissolution, or degradation) in the gel structure. In fact, the drug penetration and release behavior depend on hydrogel’s network that can easily be tuned by crosslink density [3,4]. There is a growing interest to achieve a rational design of advanced hydrogels with tunable properties. The incorporation of nanoparticles into the polymer matrix is a versatile method for controlling the crosslink density of hydrogels and drug release control. A combination therapy of nanoparticles and drugs provide an effective strategy to improve the pharmacological activity and efficacy of drugs. Moreover, the hybrid systems can be useful in long-term delivery systems [5,6,7,8,9]. On the other hand, hydrogels can provide nanoparticles with the clinically useful formulation needed for their clinical applications. The innovative combination of polymer hydrogels and nanoparticles with entirely different properties generate not only structural diversity but also create synergistic, unique, and potentially useful properties that are not found in the individual components [10,11,12]. The incorporation of metal nanoparticles into polymer network (PN) hydrogels offers an excellent alternative to control the release of nanoparticles and for applications requiring higher mechanical toughness. Interpenetrating polymer network (IPN)-based hydrogels are defined as the combination of two or more polymer networks which interpenetrate within each other at the molecular level without being covalently bonded [13,14]. These hydrogels act as templates for a variety of loads and for the synthesis of nanoparticles smaller than 2–3 nm with a uniformly distribution [15,16].

Herein, we described the preparation and characterization of new, pH-responsive, hybrid hydrogels made from polyaspartic-acid-based, interpenetrating network (IPN) hydrogel nanocomposites using 2D graphene nanosheets, nanoparticles (NPs), and acrylic copolymers as antibacterial and drug delivery systems. In the first step, polyaspartic acid hydrogel was synthesized by the combination of polysuccinimide (PSI) and GO-NH_2_ as the primary link crosslinking agents. The interaction of PSI polymers chains with GO-NH_2_ leads to the formation of a polyaspartic acid hydrogel. Polyaspartic acid (PAsp), which has a protein-like structure, is an appropriate candidate for the preparation of hydrogels because of its biodegradation and biocompatibility properties [17,18]. This polyanionic hydrogel contains ionizable groups that exhibit high sensitivity to pH variations. In the next step, acrylic monomers were polymerized onto and into the PAsp hydrogel by *N*,*N*′-methylenebisacrylamide (NMBA) as secondary link crosslinking agents. Finally, different metal nanoparticles including Ag NPs, CuO NPs, and ZnO NPs were formed inside the network by physically embedding them into the hydrogel matrix. The IPNs showed an excellent loading capacity for curcumin as well as antibacterial activity against Gram-positive and Gram-negative bacteria. The release profile of curcumin depended on the embedded metal nanoparticles and the medium conditions. Collectively, our results showed that such systems can be considered as an attractive and promising formulation for various biomedical applications such as a wound dressing, and they can generate a step forward in applications for skin.

## 2. Experimental

### 2.1. Materials

*N*,*N*′-methylenebisacrylamide (NMBA) (99%), graphite powder, N-hydroxysuccinimide (NHS) (98%), *N*,*N*’-dicyclohexylcarbodiimide (DCC) (99%), ethylenediamine (EDA) (≥99%), L-aspartic acid (≥98%), and curcumin (Cur) (≥94%) were purchased from Sigma-Aldrich (Steinheim, Gemany). Silver nitrate (AgNO_3_), zinc nitrate hexahydrate (Zn(NO_3_)_2_·6H_2_O) (98%), copper(II) chloride(CuCl_2_), acrylamide (Am) (99%), acrylic acid (AA) (98%), and all other reagents and solvents were purchased from Merck (Kenilworth, NJ, USA) and used as received. *Staphylococcus aureus* (PTCC 1112, Pasteur Institute, Tehran, Iran) and *Escherichia coli* (PTCC 1330, Pasteur Institute, Tehran, Iran) were used for the antimicrobial tests.

### 2.2. Methods

The Fourier transform infrared (FTIR) spectra of samples were recorded at room temperature using a Shimadzu FT-IR 8400 spectrometer (Kyoto, Japan) with a KBr pellet at a scanning range from 4000 to 500 cm^−1^ and a weight ratio of 5/200 mg. All UV-visible spectra were recorded at room temperature for well-dispersed nanocomposite samples in the wavelengths ranging from 400 to 800 nm using a Shimadzu UV–Vis 1650 PC spectrophotometer (Kyoto, Japan). Thermogravimetric analysis (TGA) was performed using STA409 apparatus (Linsei) from 25 °C to 900 °C at a scan rate of 10 °C/min in a nitrogenous atmosphere. X-ray diffraction (XRD, PANalytical company, Almelo, the Netherlands) patterns of hydrogels were recorded with a Siemens diffractometer using Cu-k radiation at 35 kV in the scan range of 2θ = 2° to 90°. For SEM (TESCAN company, Brno, Czech Republic) characterization, the hydrogel samples were freeze-dried and then kept overnight in a vacuum chamber to remove excess solvent. Then, the SEM pictures were taken by a LEO 440i scanning electron microscope after a gold coating. Statistical analysis was performed upon 100 nanoparticles randomly selected at 89 kX magnification. The diameter of the nanoparticles was calculated using measurement software.

### 2.3. Preparation of Poly(succinimide) (PSI)

Polysuccinimide (PSI) was synthesized through a polycondensation process according to the previously reported procedure in literature [19]. Briefly, L-aspartic acid and o-phosphoric acid (85%) in a weight ratio of 1:1 were placed into an electric thermostatic drying oven for 4.5 h at 180–200 °C. The reaction mixture was then cooled and dissolved in dimethylformamide (DMF). The resulting solution was precipitated by adding methanol as a nonsolvent, washed thoroughly with distilled water, and dried under vacuum at 65 °C for two days. (Yield 87%).

### 2.4. Synthesis of Graphene Oxide (GO)

Graphene oxide was prepared through the improved Hummers’ method [20] by the slow addition of graphite (3 g) to a mixture of H_2_SO_4_ (360 mL) and H_3_PO_4_ (40 mL). KMnO_4_ (18 g) was added to the reaction system slowly upon stirring for 1 h. Then, the reaction was heated to 50 °C and stirred for an additional 24 h. Afterwards, distilled water (400 mL) with 30% H_2_O_2_ (15 mL) was injected into the reaction system to terminate the reaction. The GO was separated by filtration and washed with 5% hydrochloric acid and distilled water. The product was dried at 40 °C for 72 h (yield 70%).

### 2.5. Synthesis of GO-NH_2_

Amine-functionalized GO was synthesized using EDA as reported in literature [21]. Briefly, NHS (11 mmol) and DCC (10 mmoL) were added into the homogeneous dispersion of GO (0.1 g) in DMF, and the mixture was stirred in a water bath at 50 °C for 1 h. Finally, EDA (50 mmoL) was added and the reaction mixture was refluxed and stirred continuously for 24 h at 80 °C to obtain amine-functionalized GO (GO-NH_2_). The suspension was centrifuged and washed several times with ethanol to remove excess EDA. The product was dried in a vacuum oven at 40 °C for 48 h (yield 85%).

### 2.6. Preparation of IPN Hydrogel

Amino-functionalized GO was used as a modifier and a crosslinking agent to interconnect the PSI chains. PSI (0.5 g) was dissolved in DMF (5 mL), followed by the addition of GO-NH_2_ (0.005 g). The reaction mixture was stirred continuously at 40 °C for 24 h. Then, the aqueous solution of monomers (14 mmol of AA and 14 mmoL of Am) was added to the above hydrogel. After nitrogen bubbled for 30 min, polymerization was carried out using NMBA (2% (*w*/*w*)) and APS (2% (*w*/*w*)) as a crosslinker and an initiator, respectively. The obtained hydrogels were placed in 50 mL of 0.1 M NaOH aqueous solution for 24 h and then immersed in water for two days to open the remaining succinimide units and to remove unreacted monomers. Finally, the prepared hydrogel was dried in a vacuum oven at 50 °C.

PAsp hydrogels were also synthesized according to the procedure described above. In short, 0.5 g of PSI was dissolved in DMF (10 mL), followed by the addition of 0.005 g of GO-NH_2_. The reaction mixture was stirred continuously at 40 °C for 24 h. The obtained hydrogels were placed in 50 mL of 0.1 M NaOH aqueous solution for 24 h, followed by an immersion in water for two days. Finally, the prepared hydrogel was dried in a vacuum oven at 50 °C.

### 2.7. Preparation of Nanoparticle-Embedded IPN Hydrogels

Due to their negative charges, hydrogels interact with metal ions electrostatically. AgNP-embedded, IPN hydrogel was synthesized according to a previously reported technique using NaBH_4_ as a reducing agent [22]. Accurately weighed, dried, IPN hydrogels were equilibrated with water for 48 h. The swollen hydrogels were immersed in a beaker containing AgNO_3_ aqueous solution with different concentrations (0.3, 0.5, and 1 mmoL) and then allowed to equilibrate for one day to entrap the silver ions (Ag^+^). Finally, in order to reduce the silver ions to silver nanoparticles, these IPN hydrogel-loaded silver salts were placed in a cold aqueous solution of NaBH_4_ for 3 h. Changing the color of the hydrogel to brown upon the reduction of silver ions confirmed the formation of silver nanoparticles (Figure S1).

The ZnO and CuO nanoparticle-embedded IPN hydrogel was synthesized using NaOH as an oxidizing agent. Typically, the swollen hydrogel was added into copper chloride and zinc nitrate solutions with different concentrations (0.000, 0.3, 0.5, and 1 mm) for 24 h. After washing with distilled water, the hydrogels were placed in 50 mL NaOH (0.1 M) solution for 24 h in order to prepare ZnO and CuO NPs. Table 1 describes the different formulations used in this work.

### 2.8. Swelling Studies

Swelling studies were conducted on IPN hydrogel and NP-embedded IPN hydrogels through the gravimetric method by the immersion of a definite amount of dried hydrogel samples in the solutions of different temperatures (25 °C–90 °C) and pH values (7.4 and 2.1). The swelling ratio (% SR) was calculated by the following equation:

SR (%) = *W*_t_ − *W*_0_/*W*_0_ × 100 (1)

where *W*_t_ was the sample weight at time t and *W*_0_ was the initial weight of the sample.

### 2.9. In Vitro Drug Loading and Release Studies

Curcumin was loaded by nanoparticle-embedded hydrogels through a swelling–diffusion approach. To load curcumin into the hydrogels, dry hydrogels (0.5 g) were soaked in 50 mL of curcumin solution (5 mg/50 mL, 2:8 ethanol:distilled water) under shaking in the dark at room temperature for 24 h. Then, the hydrogels were rinsed thoroughly with distilled water to remove the surface-adsorbed curcumin. The loading capacity was calculated according to the following Equation (2):

Drug loading efficiency (%) = *W*_e_/*W*_0_ × 100 (2)

where *W*_e_ and *W*_0_ are the weight of the drug in the hydrogel and weight of the initial drug, respectively.

The drug release profiles of curcumin-loaded hydrogels were investigated at different pHs (2.1 and 7.4) at 37 °C. The amount of released curcumin was quantified by UV/Vis spectrophotometry at 430 nm using a curcumin standard calibration curve. The percentages of drug released were calculated by the following equation:

Drug release (%) = *M*_t_/*M*_0_ × 100 (3)

where *M*_t_ and *M*_0_ are the amount of curcumin released at time and the amount of curcumin loaded into the hydrogels, respectively.

The NP release profiles of hydrogels were investigated at pH 7.4 at 37 °C. The absorbance of Ag, CuO, and ZnO NPs were recorded at 421, 280, and 349 nm wavelengths respectively, by UV/Vis spectrophotometry at different times.

### 2.10. Antibacterial Activity

The antibacterial properties of IPNs samples were evaluated by the colony-counting method (CFU) by using *Escherichia coli* (*E. coli*) Gram-negative bacteria and *Staphylococcus aureus* (*S. aureus*) Gram-positive bacteria as model organisms [23]. Monocolonies of *E. coli* and *S. aureus* were cultured on liquid luria-bertani (LB) culture medium (20 mL) and were kept in an incubator at 37 °C for 12 h. The bacteria were diluted with phosphate-buffered saline (PBS) to 10^6^–10^7^ CFU ml^−1^. Next, 20 mg of the samples was mixed with 1 mL *E. coli* and *S. aureus* bacteria solution in the dark for 30 min. Then, 150 μL of the suspension was spread on LB agar plates, and the colonies were counted after 12 h incubation at 37 °C to estimate the loss of viability of *E. coli* and *S. aureus* cells on the samples.

## 3. Results and Discussion

**Scheme 1:**
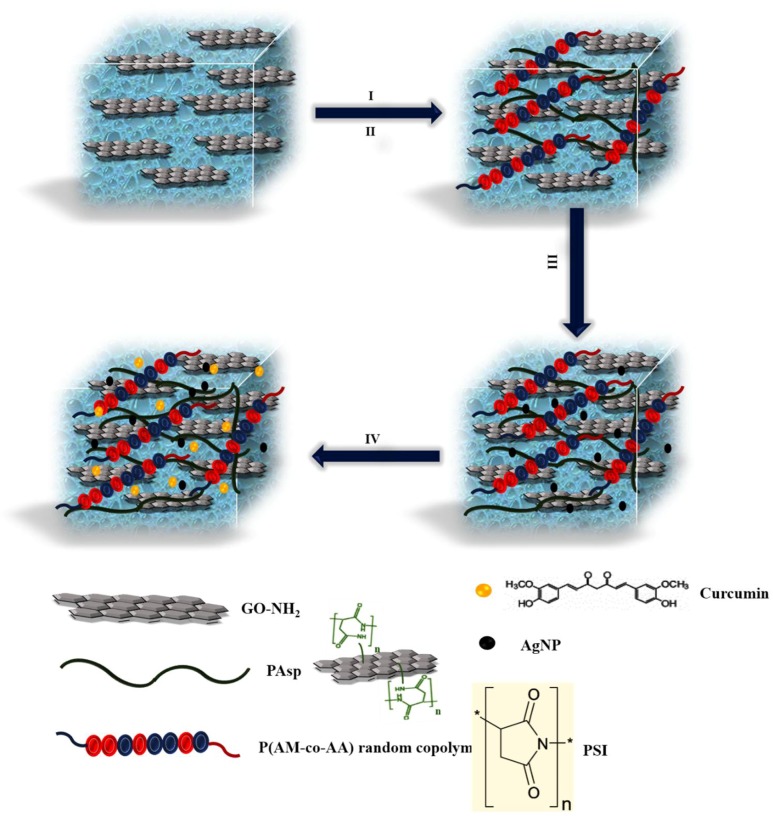
Representation of the synthesis of interpenetrating polymer network (IPN) nanoparticles (NPs). I) Reaction of the polysuccinimide (PSI) and graphite oxide (GO)-NH_2_ to the formation of a polyaspartic acid (PAsp) hydrogel, II) Polymerization of acrylic monomers onto and into the PAsp hydrogel, III) The formation of Ag, CuO, or ZnO nanoparticles inside the hydrogels, and IV) Loading of the drug inside the hydrogels.

The use of IPN hydrogels for the stabilization of nanoparticles and drug delivery is well-established and has attracted great attention in the scientific community [24]. In this context, IPN hydrogels composed of a PAsp network, acrylic polymer gel, metal nanoparticles, and graphene were prepared and used as a drug delivery method and as antibacterial systems. The synthesis of IPN hydrogel is schematically presented in Scheme 1.

The compositions and physicochemical properties of IPNs were changed by variations in the synthetic parameters (Table 1).

**Figure 1:**
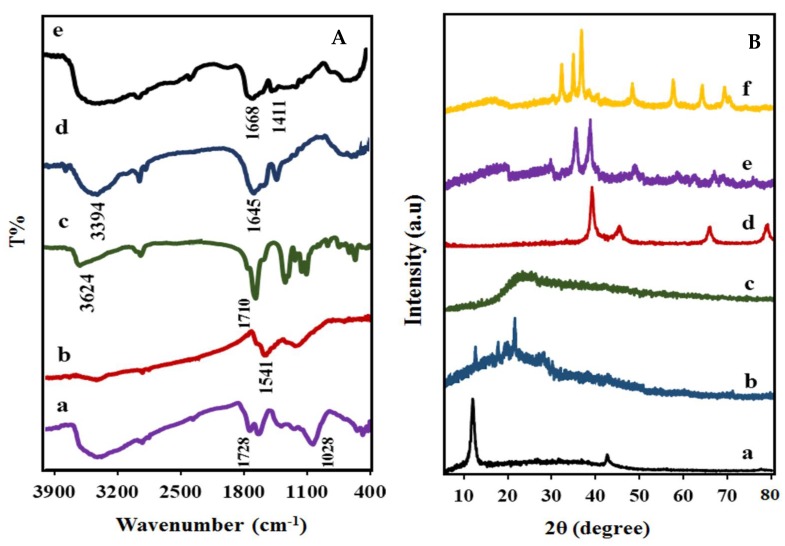
(**A**) IR spectra of (**a**) GO, (**b**) GO-NH_2_, (**c**) PSI, (**d**) PAsp hydrogel and (**e**) IPN0 hydrogel; (**B**) X-ray diffraction pattern of (**a**) GO, (**b**) GO-NH_2_ (**c**) IPN0 hydrogel, (**d**) IPN3, (**e**) IPN6, and (**f**) IPN9 NP hydrogel.

IPN hydrogels were characterized using different spectroscopy and microscopy methods as well as thermal analysis. The IR spectrum of GO (Figure 1Aa) shows stretching vibrations at 3357 cm^−1^, 1728 cm^−1^, 1627 cm^−1^, 1236 cm^−1^, and 1028 cm^−1^, which correspond to the vibration of hydroxyl, carbonyl, C=C, C–O, and C–O–C, respectively [25]. Significant changes were observed in the IR spectrum of GO upon functionalization with ethylenediamine (Figure 1Ab). The disappearance of the epoxy peak at 1028 cm^−1^ and the appearance of new peaks at 1639 cm^−1^ (assigned to amidic carbonyl groups), 1541 cm^−1^ (corresponded to N–H stretching vibrations in the C–NH group), and 1195 cm^−1^ (assigned to C–N stretching vibrations in the –C–NH–C– group) in the IR spectrum of GO-NH_2_ confirmed the successful conjugation of ethylenediamine to both the epoxide and carboxylic acid groups of GO [26]. The IR spectrum of PSI represented absorption bands at 3624 cm^−1^, 1770 cm^−1^, 1710 cm^−1^, 1394 cm^−1^, and 1361 cm^−1^ which are related to NH–, cyclic imide, –(OC)_2_N–, C–O, –(OC)_2_N–, C–N, and (C=O)–NH groups, respectively [19]. (1HNMR of PSI is shown in Figure S2. In this spectrum, the signal at 5.26 ppm corresponded to the methine proton (a) and the signals of methylene proton (b) appeared at 3.2 and 2.7 ppm. The obtained results from IR and ^1^HNMR confirmed the successful synthesis of PSI).

In comparison with the IR spectrum of PSI, PAsp hydrogel showed two new absorbance bands centered at 1645 cm^−1^ that corresponded to the vibration of the new amidic C=O groups. These new carbonyl groups were created by the reaction between GO–NH_2_ and the imide ring of PSI. The aforementioned results in combination with the disappearance of absorbance bands at 1716 cm^−1^ and 1799 cm^−1^ (assigned to the succinimide groups) in the IR spectrum of PAsp hydrogel confirmed the successful aminolysis of PSI by GO–NH_2_ [27]. The IR spectrum of IPN0 hydrogels (Figure 1Ae) indicated that this hydrogel was combined with PAm, PAA, and PAsp. Two absorbance bands around 2800 cm^−1^ and 2900 cm^−1^ (assigned to methyl groups) overlapped with an absorbance band at 3427 cm^−1^ which corresponded to the carboxyl groups. This confirmed the successful polymerization of the acrylic copolymer (AA−Am) within the PAsp hydrogel. In addition, characteristic peaks at 1668 cm^−1^ (C=O stretching of amide group), 1725 cm^−1^ (C=O stretching of carboxyl group), 1598 cm^−1^ (COO^−^ asymmetric stretching), 1411 cm^−1^ (COO-symmetric stretching mode), and 1450 cm^−1^ (C–H bending) could be observed in the IR spectra of the IPN0 hydrogels. This result proved that PAm and PAA had been successfully polymerized in the hydrogel networks [28].

XRD was used to investigate the crystal structure of materials. The XRD diagram of the GO nanosheets revealed a peak at about 2θ = 11.87°, which corresponded to the oxidation of graphite powder [21]. However, after the grafting of amino groups, the intensity of this peak was significantly reduced and another broad peak of graphite at 2θ value 20.6° appeared, which indicated a slight reduction of GO by ethylenediamine [26]. The absence of the graphene oxide diffractions in the IPN0 hydrogel and the changes in crystal structure of graphene oxide after forming the PAsp hydrogel implied a uniform dispersion of the graphene sheets in the hydrogel (Figure 1Bc). This was due to a reaction between the amino groups of GO–NH_2_ and the succinimide units of the PSI chains. Also, the broad peaks observed in the IPN0 hydrogel (Figure 1Bc) at 22° were assigned to the polymeric matrix. The embedding of metal nanoparticles into the IPN hydrogels was evaluated by XRD. Figure 1B shows the X-ray powder diffraction pattern of the IPN3, IPN6, and IPN9 NP hydrogels. The sharp peaks in IPN NP hydrogels, indicating the crystalline character of the materials, can be assigned to reflections from characteristic crystal planes of the NPs. IPN3 hydrogel exhibited sharp and intense peaks at 38.35°, 44.54°, 64.75°, and 77.63° (Figure 1Bd), which were attributed to (111), (200), (220), and (311) Bragg’s reflections of the silver nanoparticles, respectively [29,30]. The XRD pattern of CuO NPs displayed characteristic peaks at 2θ of 29.41°, 34.95°, 38.11°, 48.12°, 57.87°, 61.48°, 65.64°, 67.72° and 74.94°C corresponding to (110), (002), (111), (102), (113), (311), (113), (311), and (004) planes of CuO NPs, respectively (Figure 1Be) [31,32].

In addition, the XRD pattern of the ZnO NPs displayed characteristic peaks at 2θ of 31.76°, 34.37°, 36.13°,47.42°, 56.63°, 62.91°, and 67.95° assigned to (100), (002), (101), (102), (110), (103), and (112) planes of zinc oxide (Figure 1Bf) [33,34]. These results confirmed the formation of crystalline NPs into IPN hydrogels. The sharp peaks indicated the crystalline character of the material. Also, the broad diffraction observed in the XRD patterns of IPN1, IPN4, and IPN7could be completely attributed to the amorphous nature of the polymer chains (Figure S3). The formation of NPs within the hydrogel networks was further investigated by UV–Vis analysis. IPN1, IPN4, and IPN7 exhibited significant absorption peaks centered at 421, 280, and 349 nm wavelengths due to the surface plasmon resonance of Ag, CuO, and ZnO NPs, respectively. Moreover, a small hump at 280 nm in the absorption spectrum of IPN1 could be attributed to ionic silver or charged clusters. This result confirmed the presence of embedded metal nanoparticles in the structure of hydrogels and is in agreement with previously published papers [35,36,37].

**Figure 2:**
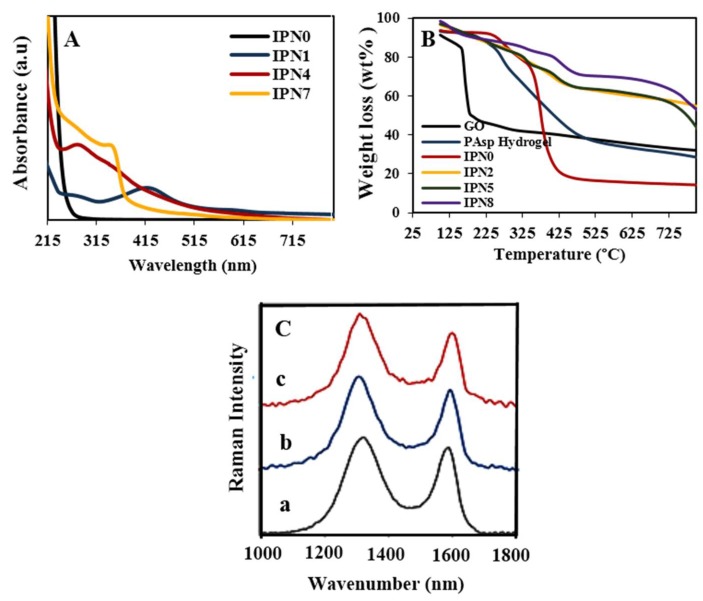
(**A**) UV-visible spectral analysis of IPN0 hydrogel, IPN1, IPN4, IPN7; (**B**) TGA thermograms of GO, PAsp hydrogel, IPN0, IPN2, IPN5, and IPN8 NP hydrogel; (**C**) Raman spectra of (**a**) GO, (**b**) GO-NH_2_, and (**c**) PAsp hydrogel.

The thermal behavior of GO and the synthesized hydrogels was investigated by TGA (Figure 2B). The initial weight loss at temperatures less than 100 °C was attributed to the evaporation of entrapped water in GO and the hydrogels. GO was thermally unstable and mainly showed mass loss (~45%) at 200 °C, which is ascribed to the removal of H_2_O and the pyrolysis of labile oxygen containing functional groups [38]. This result showed that the prepared GO was strongly oxidized. The decomposition onset temperature of GO increased to 220 °C and weight loss was mainly observed at 220–500 °C when it was embedded in PAsp hydrogel. The higher thermal stability of GO in the hydrogel was due to a slight reduction of sheets by ethylenediamine and their crosslinking by PSI. The temperature of the pyrolysis of the IPN0 hydrogel increased, and it revealed two major weight losses in the range of 250–360 °C and 360–460 °C which were due to the decomposition of the PAsp hydrogel and the acrylic copolymer chains, respectively. On the contrary, the maximum decomposition temperatures (Tmax) of the hydrogels, which were determined from the peak temperature in the differential thermogravimetric (DTG) curves, were 280, 320, 412, 415, and 420 for the PAsp hydrogel, IPN0, IPN2, and IPN8, respectively (Figure S4). These results indicated that the thermal stability of the hydrogel was increased by the incorporation of NPs. Moreover, the high char yield, which was up to 48.8, 27.5, and 33.3 wt % at 900 °C, was observed in the thermogram of the IPN2, IPN5, and IPN8 hydrogels, respectively. This could be attributed to the metal nanoparticles inside the hydrogels.

Raman spectroscopy provides a powerful and nondestructive method to investigate the structure and integrity of carbon-based nanomaterials. The Raman spectra of GO, RGO–NH_2_, and the PAsp hydrogel are shown in Figure 2C. The spectrum of GO showed peaks at 1587 cm^−1^ and 1322 cm^−1^; these are called G bands, which originated from the first-order scattering of the E2g phonons of the sp^2^-hybridized carbon atoms, and D bands, which are attributed to the k-point phonons of A1g symmetry of the defects involved in the sp3-hybridized carbon bonds, respectively. The intensity ratio of D band to G band (I_D_/I_G_) as a measure of the graphitization degree of carbon materials is usually used to determine how a modification disrupts the structure of graphite. The G and D mode of RGO–NH_2_ showed blue and red shifts to 1590 cm^−1^ and 1309.20 cm^−1^, respectively. A shift in G peak and a slight increase in the D/G-ratio of GO–NH_2_ (1.16) compared to GO (1.15) suggests that the sp^2^ structure of graphene sheets was destructed to a high extend. These defects could be attributed to the restoration of sp^2^ as new graphitic domains with a smaller size than the ones present in GO [21]. Also, when GO–NH_2_ was embedded in PAsp, the G-band shifted to 1598 cm^−1^ and the I_D_/I_G_ ratio increased (1.25) in comparison with GO–NH_2_ (1.16). This result was due to the increase in the number of sp^3^ carbons upon chemical functionalization. These results in addition to the appearance of an additional weak shoulder at 1340 cm^−1^, which was assigned to PAsp moieties, confirmed the grafting of the PAsp on the GO–NH_2_ surfaces.

**Figure 3:**
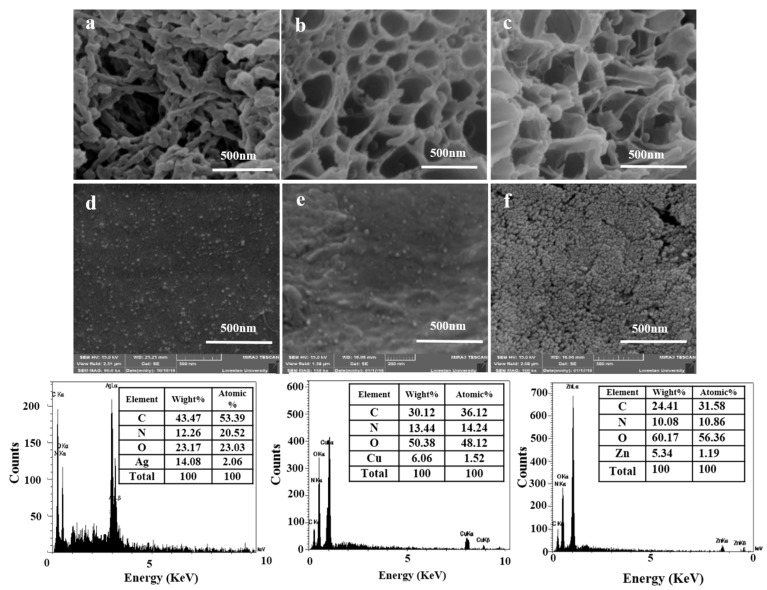
SEM images of (**a**) PAsp hydrogel; (**b**,**c**) IPN0 hydrogel; SEM images and EDX diagram (**d**) IPN2; (**e**) IPN4; (**f**) IPN7 hydrogel.

SEM measurements were used to evaluate the surface and interior morphology of synthesized hydrogels. The SEM images of PAsp, IPN, and three IPN NP hydrogels are shown in Figure 3. A network structure with a large number of pores and an average size of less than 500 nm in length was observed in the PAsp hydrogel. These pores are attributed to the formation of a 3D network in PSI by GO–NH_2_ as a crosslinking agent (Figure 3a). The IPN0 hydrogel showed a uniform, porous structure with an approximate pore size (e.g., 200 ± 8 nm) which would be suitable for the formation of nanoparticles and drug loading (Figure 3b,c). The difference between the morphology of PAsp and IPN0 hydrogels probably originated from the interactions between the networks during the polymerization processes. SEM images further revealed the formation of nanoparticles in the IPN hydrogels. SEM images of the IPN NP hydrogels showed a homogeneous distribution of the NPs through the hydrogel matrix when the swollen hydrogels were placed into the silver nitrate, Zn nitrate solution. Aggregations or clusters of NPs were not observed because they stabilized through the interfacial interactions with the carboxyl groups of the hydrogel matrixes. During this process, most of the ions were loaded into free-network spaces of the hydrogel. Also according to the results of the SEM images (Figure S4), almost no distinguishable difference was found in the NP size for the hydrogels with different contents of AgNO_3_, CuCl_2_, and Zn(NO)_3_·6H_2_O. This is probably due to the optimization of the amount of polysuccinimide (PSI) and the acrylic acid monomer (Figure S5). Energy Dispersive X-Ray Spectroscopy (EDX) analysis showed peaks at 3, 1, and 1 keV for the IPN NP hydrogels (Figure 3) that corresponded to the AgNPs, CuONPs, and ZnONPs and their surface plasmon resonance. This is in agreement with previously published papers [39,40,41].

**Figure 4:**
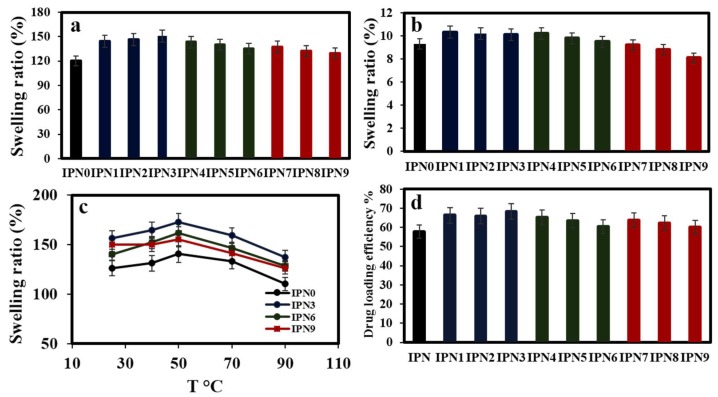
Swelling behavior of different hydrogels at 25 °C and (**a**) pH 7.4 and (**b**) pH 2.1; (**c**) Swelling behavior of IPNc hydrogels versus temperature at pH 7.4; (**d**) Loading efficiency of hydrogels for curcumin.

The swelling behavior of hydrogels is a crucial factor to evaluate their capability to absorb and release drugs [42]. Therefore, the swelling behaviors of the synthesized hydrogels were investigated in various environmental conditions (Figure 4 and Figure S6). The hydrogels showed similar swelling profiles. Figure S7 shows the swelling behavior of IPNs as a function of time. The plotted curves in Figure S6 showed that IPNs have similar swelling behavior with a relatively slower saturation after 8 h. In order to study the effect of the pH of the medium on the swelling characteristics of hydrogels, the swelling test of the IPN hydrogels before and after the formation of NPs was accomplished at pH 2.1 and 7.4 at 37 °C (Figure 4a,b). At pH 2.1, the swelling ratios were not significant for all hydrogels. This is attributed to the formation of hydrogen bonds between carboxyl, amine, and amide bonds in the hydrogels, which reduced their interaction with water molecules. However, at pH 7.4, the swelling ratio significantly increased in the first 30 min of immersion into water and reached a maximum constant swelling after 8–12 h. This is due to the repulsion between negatively charged carboxyl groups at neutral pH, causing bigger pore sizes. Most significantly, IPN NP hydrogels exhibited a higher water-absorbing capacity than the IPN0 hydrogel at pH 7.4, probably due to the decreased crosslink density and enlargement of the hydrogel networks in the presence of NPs which resulted in an increased degree of hydration and higher water penetration. The formation of nanoparticles in the hydrogel networks may have caused an increase in the pore size and space inside the hydrogel (Figure S8) [43,44,45]. The swelling curves of the hydrogels at different temperatures (25–90 °C) are also shown in Figure 4c. There was a direct correlation between the swelling ratios of the IPN hydrogels and the environmental temperature at a 25–60 °C range. However, temperatures higher than 60 °C caused the swelling ratios to be decreased. This is due to the collapse of the hydrogel pores at higher temperatures.

**Figure 5:**
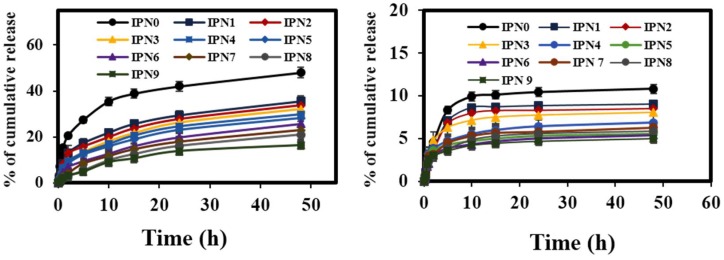
Release profile of curcumin from IPNs (**a**) pH 2.1 and (**b**) 7.4.

Because of their pH-responsive behaviors, the synthesized hydrogels are promising candidates for drug delivery. Accordingly, the ability of the synthesized IPN hydrogels for the controlled delivery of curcumin was investigated. Curcumin was loaded by hydrogels using a swelling diffusion approach. Hydrogels with the loaded curcumin are abbreviated as IPN@Cur. The effect of NPs on the drug loading efficiency of the IPN hydrogels is summarized in Figure 4d. All the hydrogels with the embedded NPs showed the highest loading efficiency for curcumin. Interestingly, the loading efficiency of IPN hydrogels increased after improving their Ag NPs content. The presence of AgNPs inside the hydrogels resulted in holes and consequently the expansion of the hydrogel network and a higher diffusion of drug [6,7]. In contrary to this result, the loading capacity of the IPN hydrogel decreased with increased ZnO NPs and CuO NPs contents. In fact, a high content of these nanoparticles inside the hydrogels led to the lower swelling of the IPN hydrogel and consequently a lower loading of drug. The release of curcumin from hydrogels with different NPs contents was evaluated at pH 2.1 and pH 7.4 and at 37 °C. As shown in Figure 5a,b, the release rate of curcumin decreased with an increased NPs content. The incorporation of nanoparticles into the hydrogels increased the stability of the loaded curcumin in the networks and led to a slower release of the drug. The release behaviors of curcumin from the synthesized hydrogels were also pH-responsive. The release of the drug from each hydrogel was maximized and minimized at pH 7.4 and pH 2.1, respectively (Figure 5). These release characteristics could be ascribed to the high compression of the hydrogel network at pH 2.1 that limits the release of curcumin from the hydrogels. The release of curcumin was increased at its natural pH due to the increased electrostatic repulsions and the reduction of hydrogen bonds between ionic groups. The release behavior was similar to the swelling ratio pattern and suggested that the pore size decreased at pH 2.1.

The mechanism of drug release from the hydrogels at pH 7.4 was evaluated using a Korsmeyer–Peppas model using the equation:

M_t_/M_∞_ = K^n^ (4)

where M_t_ and M_∞_ are the amount of the drug released at time t and at infinite time, respectively, and K and n are the release rate constant and the release exponent, respectively.

The values of K and ‘n’ along with the values of the correlation coefficient, r, are shown in Table 2. The K values decreased in the presence of nanoparticles, especially ZnO NPs. The K values ranging from 0.1 to 0.01 indicated the prolonged release of curcumin from the hydrogels. These values suggested that the release of drug from the hydrogels is diffusion-controlled and approximately close to the Korsmeyer–Peppas model [42,46]. The role of graphene sheets, which show a high π–π interaction with curcumin, in the prolongation of drug release should not be ignored [46].

**Figure 6:**
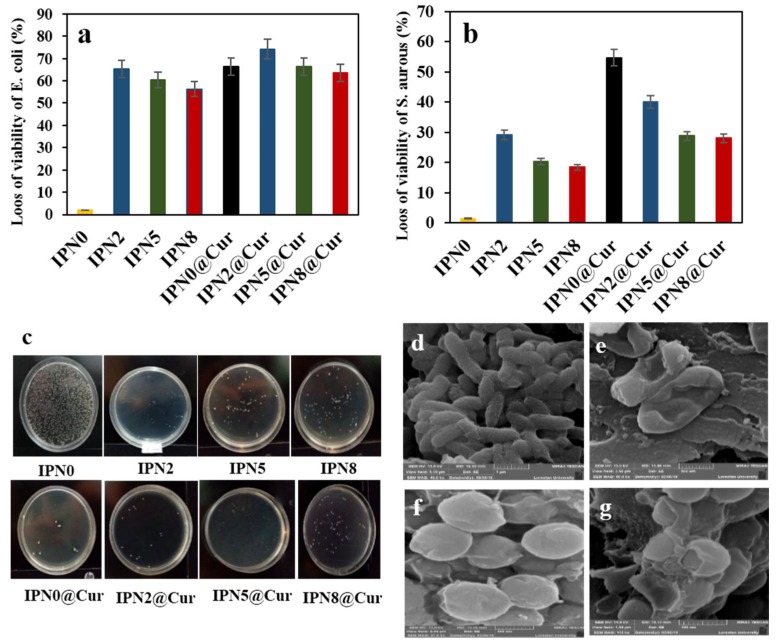
The viability of (**a**) *E. coli* cells (10^6^−10^7^ CFU/mL) and (**b**) *S. aureus* cells (10^6^−10^7^ CFU/mL) incubated with hydrogels (2 mg) for 12 h; (**c**) Photograph of *E. coli* colonies grown on agar plates after exposure to IPN hydrogels. SEM images of *E. coli* after incubation with (**d**) saline solution and (**e**) IPN2@Cur for 12 h. SEM images of *S. aureus* after incubation with (**f**) saline solution and (**g**) IPN2@Cur for 12 h.

The antibacterial activity of hydrogels against Gram-positive bacteria (*S. aureus*) and Gram-negative (*E. coli*) was examined after exposing the bacteria cells (10^6^ to 10^7^ CFU/mL) to the same amount (20 mg) of IPN hydrogels in isotonic saline solution for 12 h under 250 rpm shaking speed. The antibacterial activity of the IPN hydrogels was evaluated using the colony count method (Figure 6). The viability of *E. coli* cells decreased by 12.3%, 67.3%, 60.4%, 56.4%, 65.3%, 74.21%, 66.3%, and 62.5%, and the viability of *S. aureus* cells decreased by 10.3%, 29.21%, 20.25%, 18.51%, 55.7%, 40.12%, 28.8%, and 28.07% after incubating them with IPN0, IPN2, IPN5, IPN8, IPN@Cur, IPN2@Cur, IPN5@Cur, and IPN8@Cur aqueous suspensions, respectively. Clearly, IPN NPs and IPN NPs@Cur show higher antibacterial activity against Gram-negative than Gram-positive bacteria. The antibacterial activity of hydrogels against Gram-negative bacteria could be due to the stronger electrostatic interactions between positively charged metal ions and this type of bacteria [31].

As expected, bacteriostatic behavior was not observed for the pure IPN hydrogels, and colonies easily formed on the pure hydrogel surfaces. However, there was a bacterial growth reduction caused by hydrogels containing NPs. Graphene oxide as the platform for growing metal nanoparticles and stabilizing them due to its large specific surface and area lateral dimension, could increase the availability of bacteria on the antibacterial materials by the adsorption of the bacteria species. IPN containing Ag NPs exhibited higher antimicrobial effects against *E. coli* than those with embedded ZnO and CuO NPs. Most significantly, the immobilization of curcumin also is further expected to enhance such activities in the IPN0 and IPN NP hydrogels. These results were expected because curcumin has shown antimicrobial activity against a wide range of bacteria [47,48]. Surprisingly, the antibacterial activity of the synthesized hydrogels containing curcumin were in line with the drug release trend. In other words, the antibacterial activity of the hydrogels containing drugs was affected by the release rate of the drug. IPN0 hydrogels showed the greatest effect of the drug against bacteria due to the high release of curcumin. In fact, depending on the speed of drug release and NP release (Figure S9), hydrogels containing nanoparticles and curcumin with a synergistic effect showed a different activity on antibacterial activity. To further investigate the antibacterial behavior of IPN@Cur, the morphology of bacteria incubated with this hydrogel was investigated by field emission (FE)-SEM (Figure 6). After treatment with IPN2@Cur for 12 h, the shape of bacteria changed significantly and their membrane was damaged. Clearly, the toxicity of the synthesized hydrogels was due to the synergistic effect of the NPs, graphene, and curcumin [49,50].

## 4. Conclusions

In summary, hybrid hydrogels consisting of IPN, PAsp, graphene, and metal nanoparticles were synthesized and their antibacterial activity and drug delivery application was investigated. Results showed that NPs have a significant effect on the loading capacity and antibacterial activity of the synthesized hydrogels. Furthermore, the antibacterial activity of the hydrogels depended on the type of metal nanoparticle, the rate of release of curcumin, as well as the type of bacteria. Due the synergic effects of different components, the synthesized hydrogels could be used for a wide range of biomedical applications.

## Figures and Tables

**Table 1:** Compositions of different IPN hydrogels synthesized and investigated in this work.

Formulation Code	Am (mmoL)	AA (mmoL)	PSI (g)	AgNO_3_ (mmoL)	CuCl_2_ (mmol)	Zn(NO_3_)_2_·6H_2_O (mmoL)
IPN0	0.05	0.06	0.1	—	—	—
IPN1	0.05	0.06	0.1	0.3	—	—
IPN2	0.05	0.06	0.1	0.5	—	—
IPN3	0.05	0.06	0.1	1	—	—
IPN4	0.05	0.06	0.1	—	0.3	—
IPN5	0.05	0.06	0.1	—	0.5	—
IPN6	0.05	0.06	0.1	—	1	—
IPN7	0.05	0.06	0.1	—	—	0.3
IPN8	0.05	0.06	0.1	—	—	0.5
IIPN9	0.05	0.06	0.1	—	—	1

**Table 2:** Drug release data of the hydrogels by Korsmeyer–Peppas (K–P) at pH 7.4.

Formulation Code	% Drug Loading	K, h^nr^	n	R^2^
IPN0	57.7	0.1023	0.478	0.999
IPN1	67.3	0.0403	0.740	0.998
IPN2	65.3	0.401	0.711	0997
IPN3	70.1	0.3721	0.955	0.997
IPN4	67.2	0.03372	0.938	0.997
IPN5	64.45	0.03483	0.933	0.997
IPN6	60.3	0.03167	1.051	0.997
IPN7	65.3	0.01702	1.257	0.998
IPN8	62.3	0.01661	1.303	0.997
IPN9	60.01	0.01521	1.330	0.998

## Supplementary Materials

The following supplementary materials are available online at http://www.mdpi.com/2073-4360/10/6/660/s1.
