# Supplementary material for: pH-Responsive Hybrid Hydrogels as Antibacterial and Drug Delivery Systems
Source: Polymers (Basel). 2018 Jun 13;10(6):660. doi: 10.3390/polym10060660 (PMC6404117; doi:10.3390/polym10060660)
Supplement: Supplementary file 1 [file polymers-10-00660-s001.pdf]

# Supplementary material

# pH-Responsive Hybrid Hydrogels as Antibacterial and Drug Delivery Systems

Shabnam Sattari, Abbas Dadkhah Tehrani, Mohsen Adeli*

Department of Chemistry, Faculty of Science, Lorestan University, Khorram Abad, Iran

E-mail: aadeli@fu-berlin.de

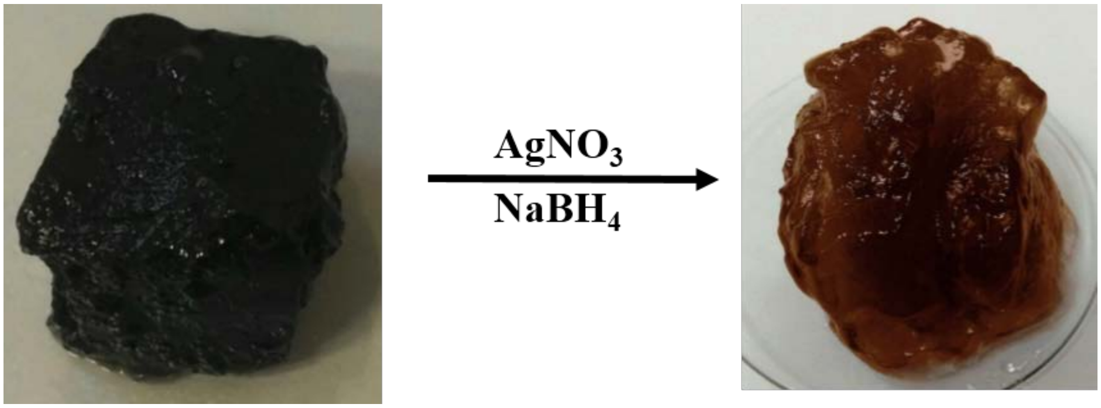


Figure S1: Photographs of before and after the formation of silver nanoparticles of silver nanoparticles

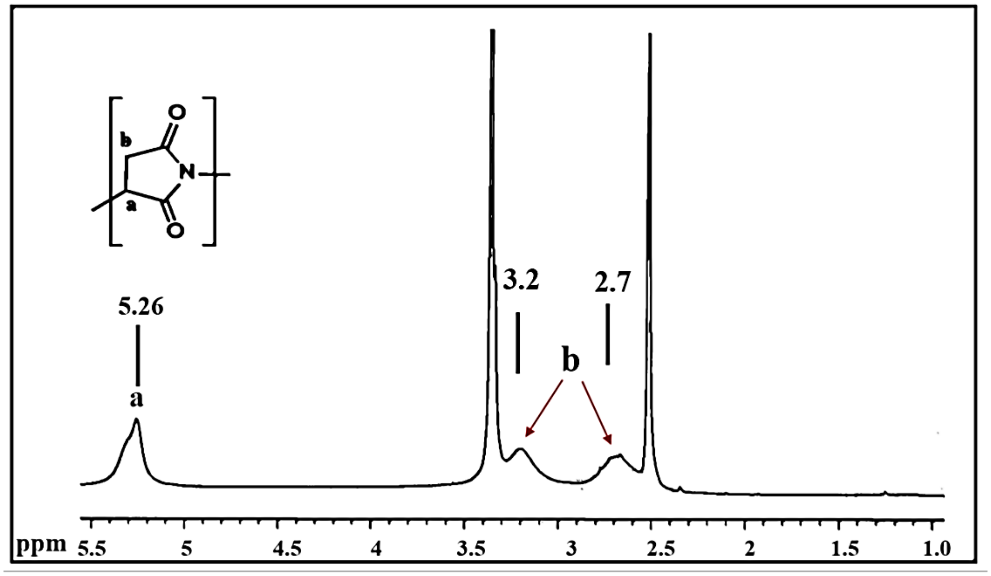


Fig. S2. 1H NMR spectra of PSI.

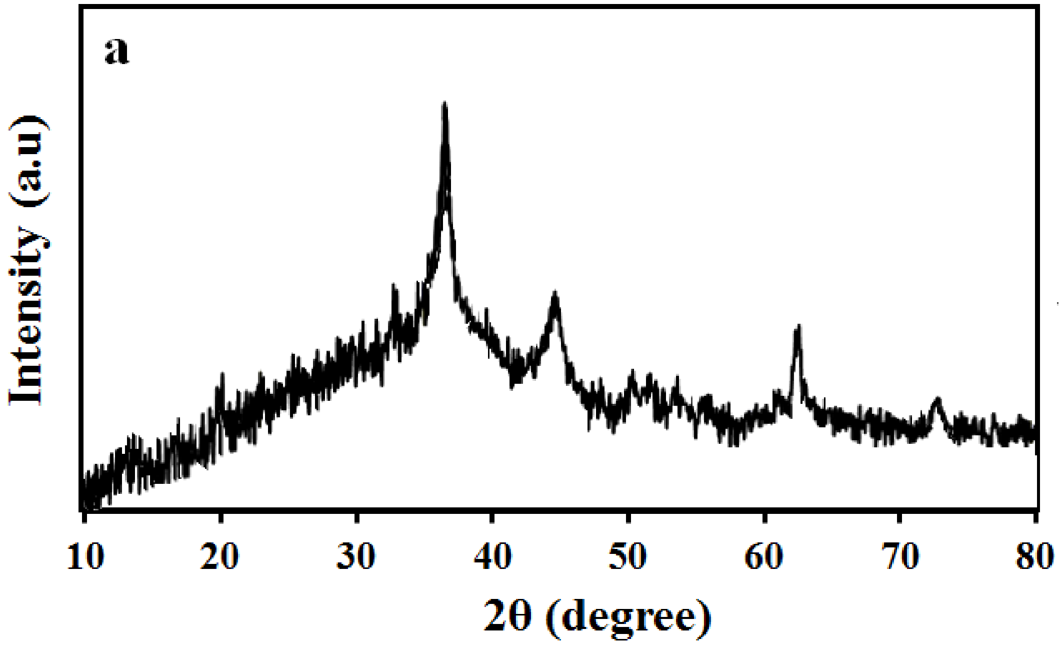

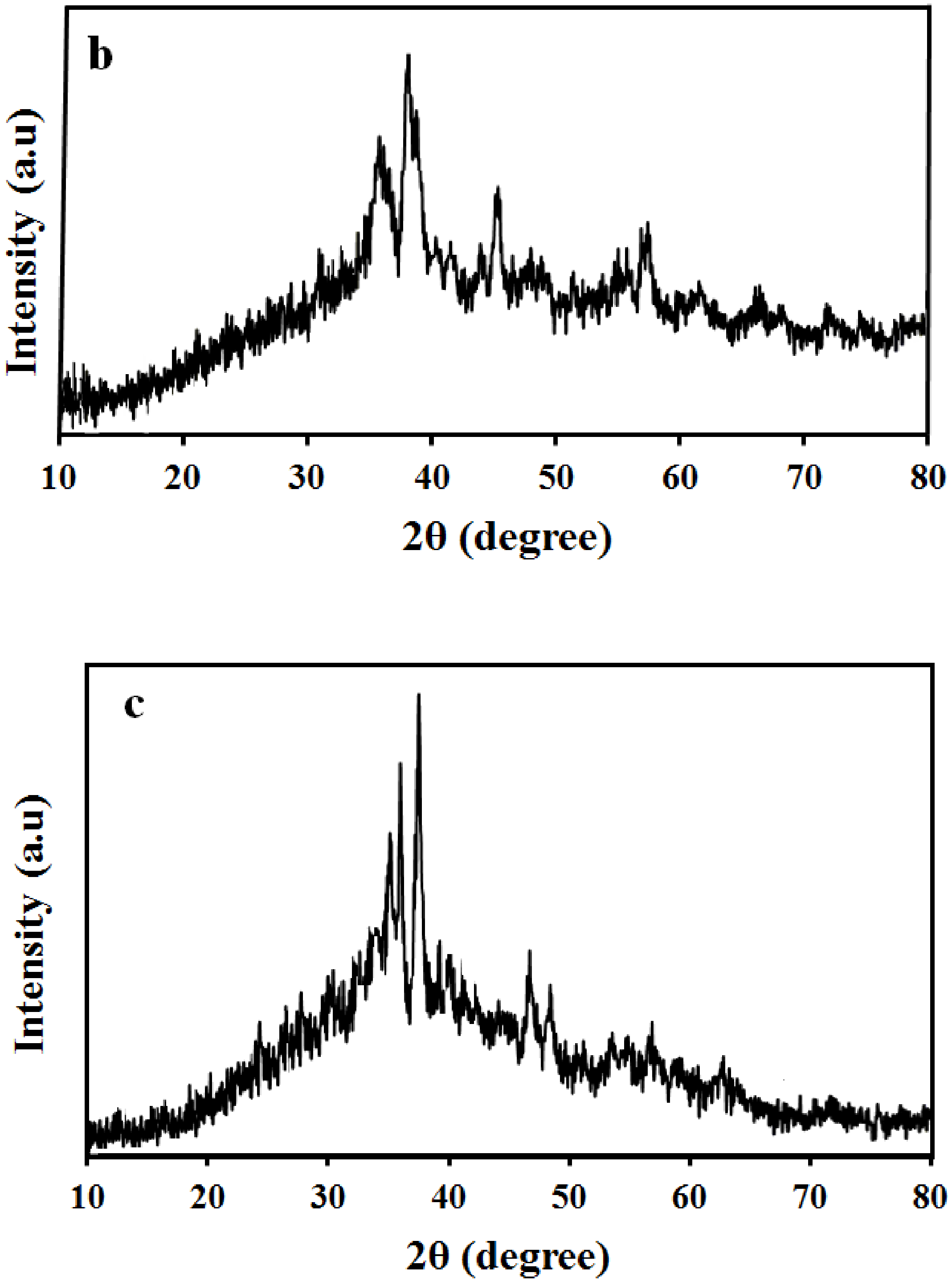


Figure S3: X-ray diffraction pattern of a) IPN1, b) IPN4 c) IPN7 NPs hydrogel.

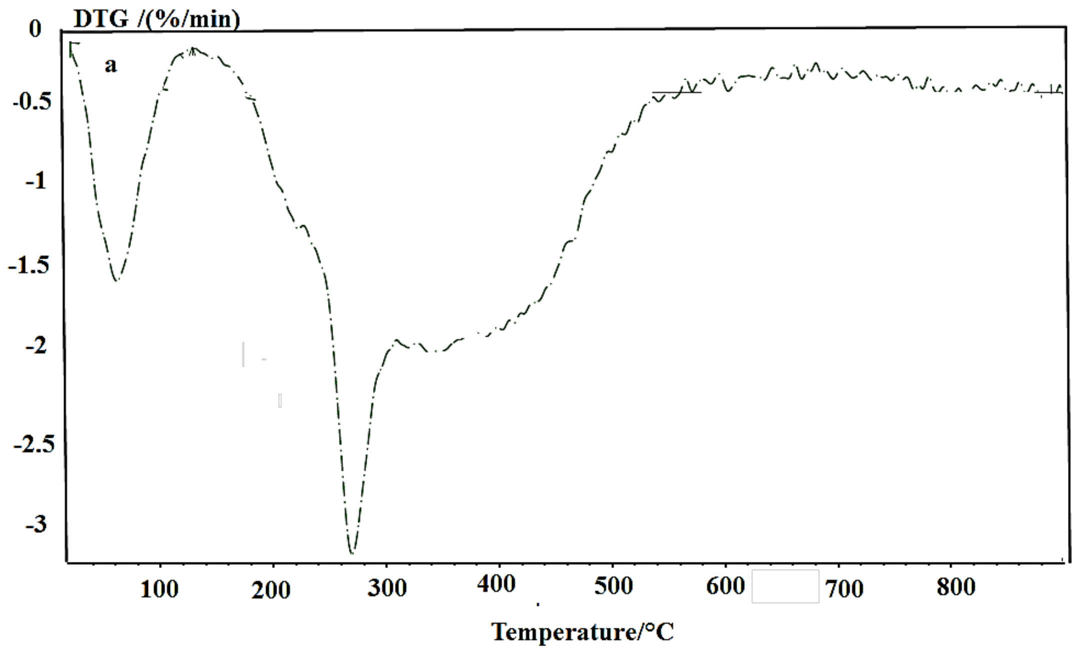
DTG /(%/min)
0
-0.5
-1
-1.5
-2
-2.5
-3
a
100
200
300
400
500
600
700
800
Temperature/°C

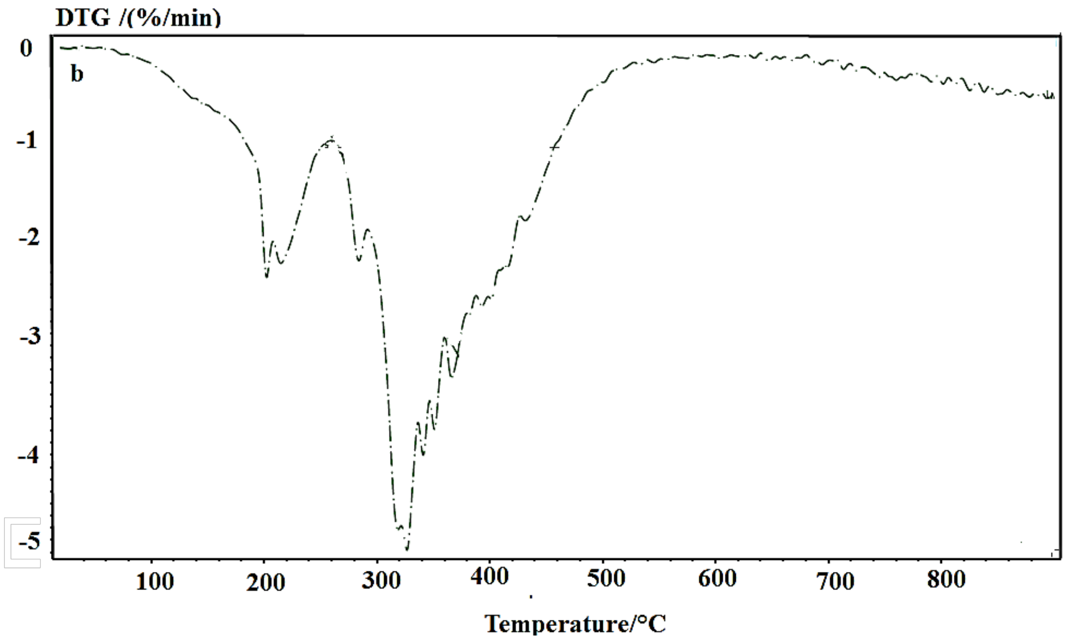
DTG /(%/min)
0
-1
-2
-3
-4
-5
b
100
200
300
400
500
600
700
800
Temperature/°C

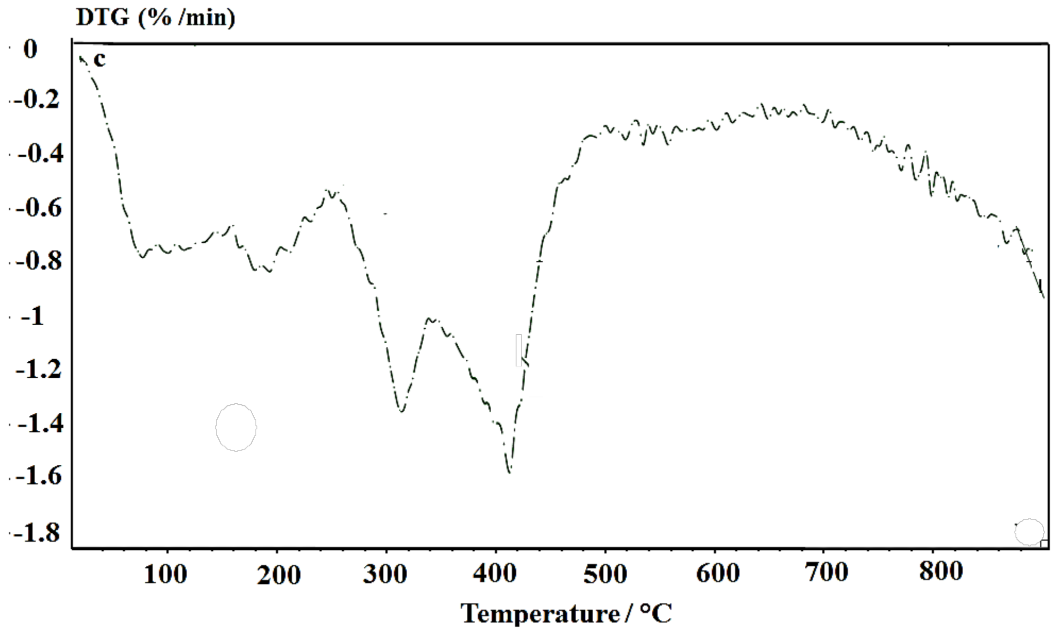

DTG (% /min)
0
-0.2
-0.4
-0.6
-0.8
-1
-1.2
-1.4
-1.6
-1.8
c
100
200
300
400
500
600
700
800
Temperature / °C


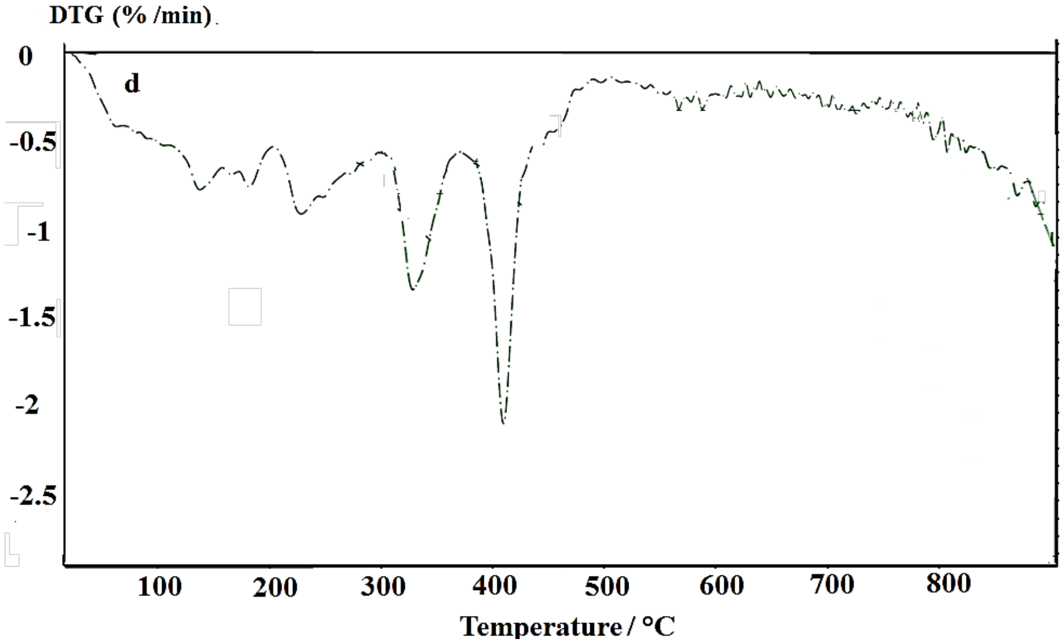

DTG (% /min)
0
-0.5
-1
-1.5
-2
-2.5
d
100
200
300
400
500
600
700
800
Temperature / °C

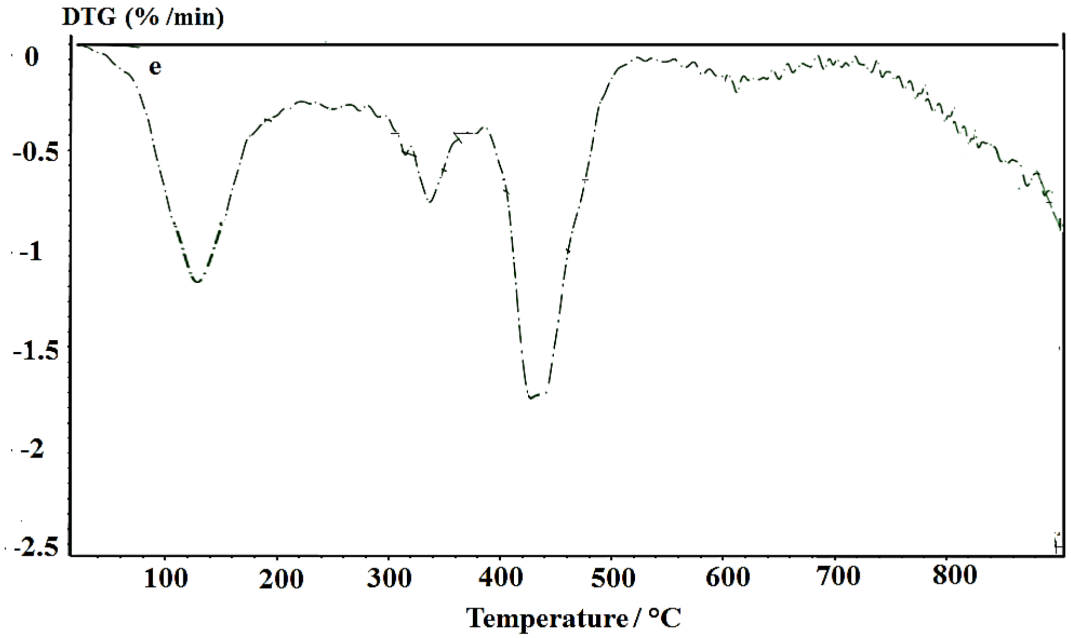


Figure S4. DTG thermograms of a) PAsp hydrogel, b) IPN0, c)IPN2, d)IPN5 and e) IPN8

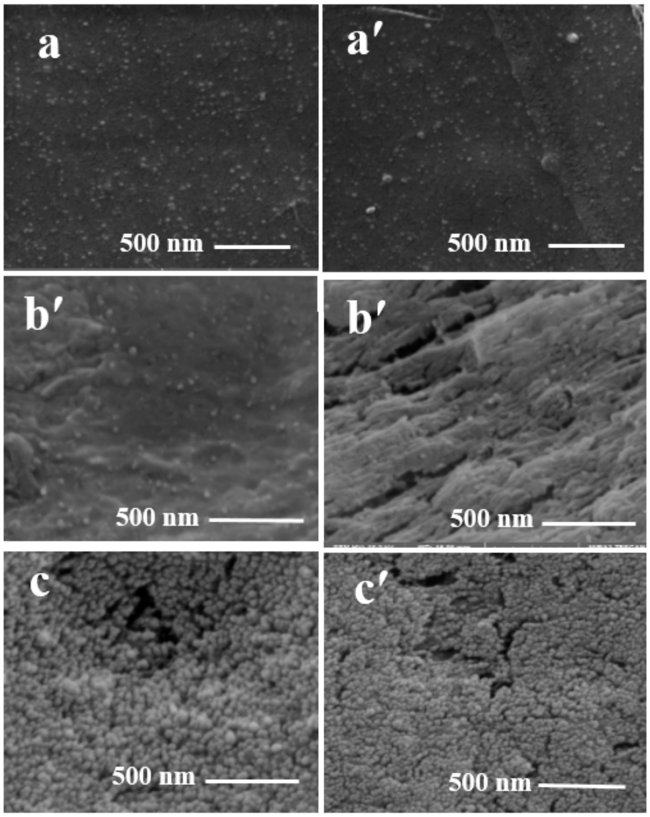


Figure S5: SEM images of a) IPN2, a) IPN3 b)IPN4, b)IPN6, c)IPN8, c)IPN9

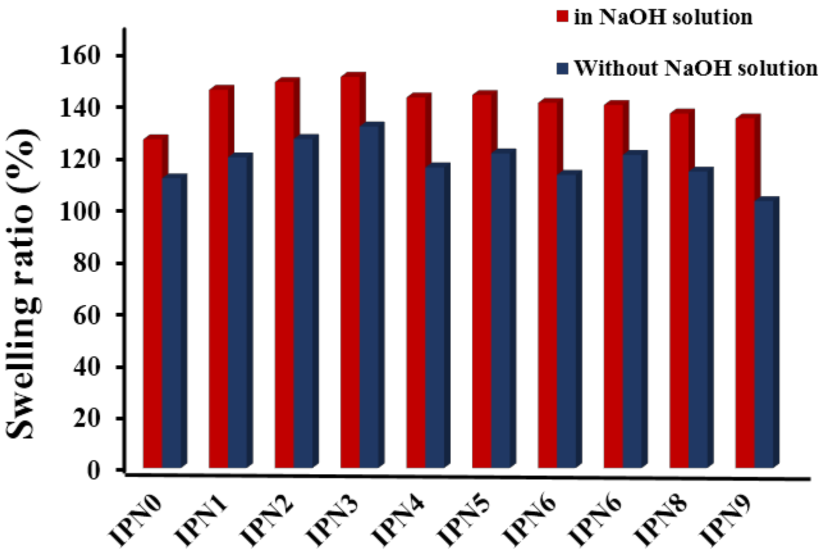

Figure S6: Swelling behavior of different hydrogels at 25°C, before and after placement of hydrogels in a NaOH solution.

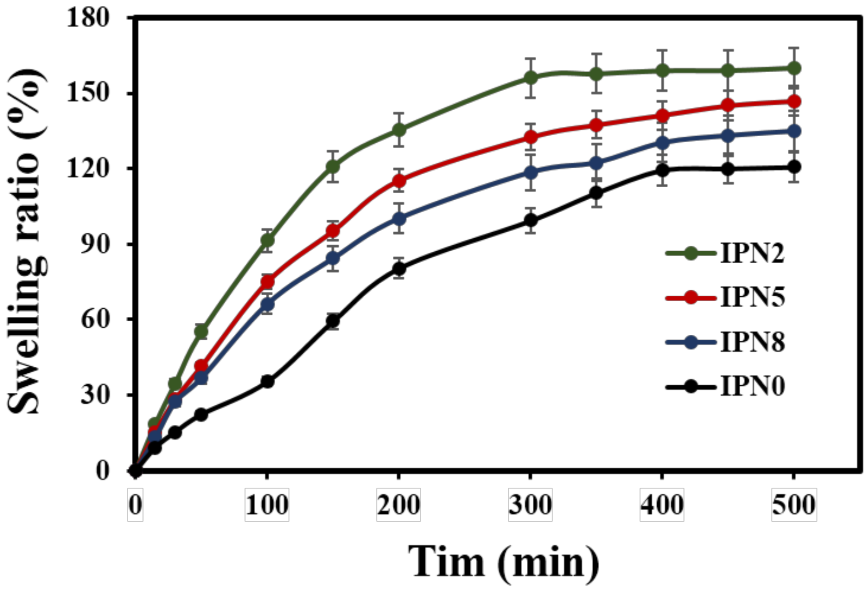


Figure S7: Swelling of the hydrogels IPNs and IPNs NPs hydrogels at pH 7.4.

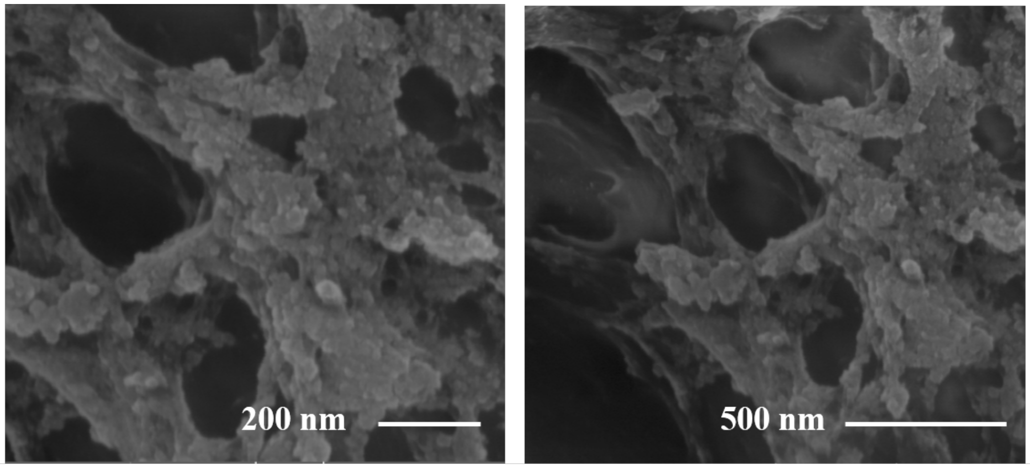


Figure 8. SEM images of IPN5 hydrogel in two different magnifications

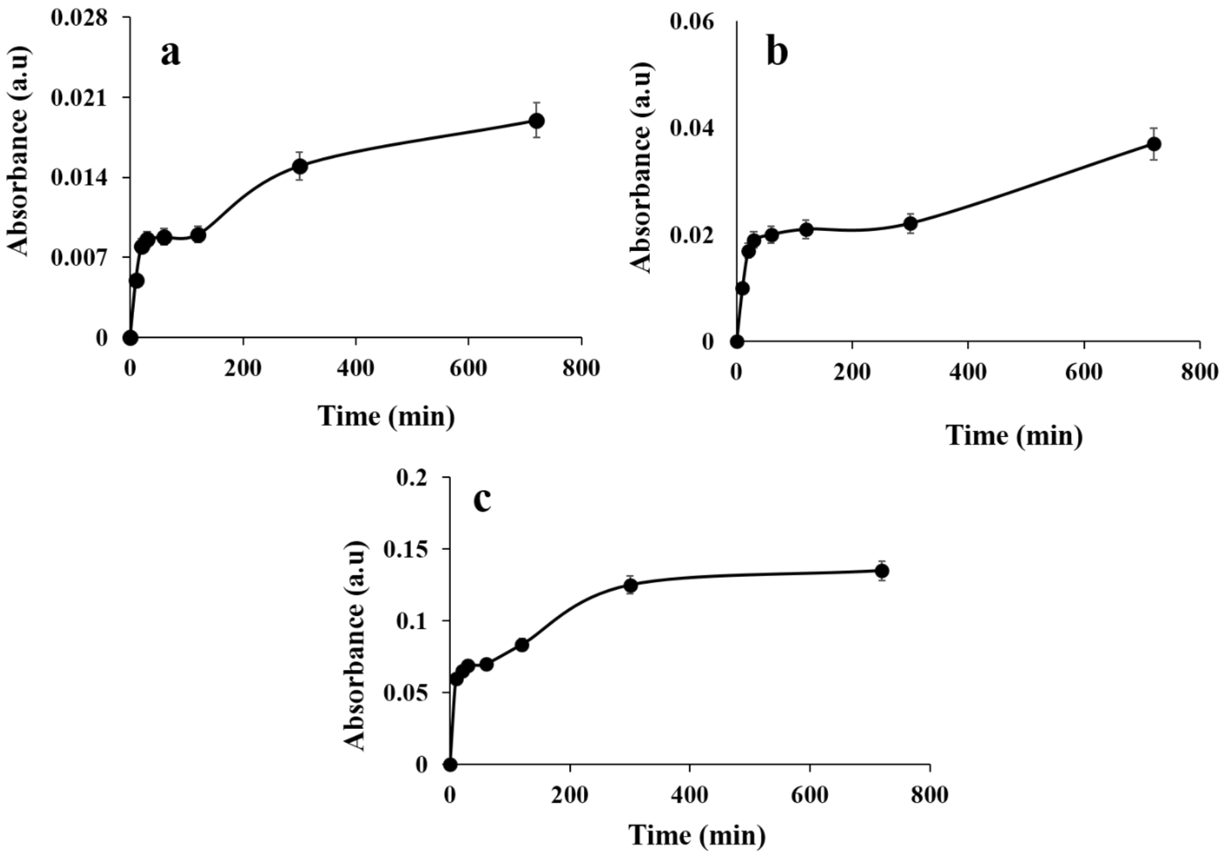


Figure S9. The release of nanoparticles from a) IPN1, b) IPN4 and c) IPN7 hydrogel.
